# Retinal Arteriolar Morphometry Based on Full Width at Half Maximum Analysis of Spectral-Domain Optical Coherence Tomography Images

**DOI:** 10.1371/journal.pone.0144437

**Published:** 2015-12-09

**Authors:** Yu Hua Tong, Tie Pei Zhu, Ze Lin Zhao, Hai Jing Zhan, Fang Zheng Jiang, Heng Li Lian

**Affiliations:** 1 Quzhou People's Hospital, Quzhou Central Hospital of Zhejiang Chinese Medical University, Quzhou, Zhejiang Province, China; 2 Eye Center of Affiliated Second Hospital, Medical College of Zhejiang University, Hangzhou, Zhejiang Province, China; 3 Eye Hospital of Wenzhou Medical University, Zhejiang Eye Hospital, Wenzhou, Zhejiang Province, China; University of Melbourne, AUSTRALIA

## Abstract

**Objectives:**

In this study, we develop a microdensitometry method using full width at half maximum (FWHM) analysis of the retinal vascular structure in a spectral-domain optical coherence tomography (SD-OCT) image and present the application of this method in the morphometry of arteriolar changes during hypertension.

**Methods:**

Two raters using manual and FWHM methods measured retinal vessel outer and lumen diameters in SD-OCT images. Inter-rater reproducibility was measured using coefficients of variation (CV), intraclass correlation coefficient and a Bland-Altman plot. OCT images from forty-three eyes of 43 hypertensive patients and 40 eyes of 40 controls were analyzed using an FWHM approach; wall thickness, wall cross-sectional area (WCSA) and wall to lumen ratio (WLR) were subsequently calculated.

**Results:**

Mean difference in inter-rater agreement ranged from -2.713 to 2.658 μm when using a manual method, and ranged from -0.008 to 0.131 μm when using a FWHM approach. The inter-rater CVs were significantly less for the FWHM approach versus the manual method (P < 0.05). Compared with controls, the wall thickness, WCSA and WLR of retinal arterioles were increased in the hypertensive patients, particular in diabetic hypertensive patients.

**Conclusions:**

The microdensitometry method using a FWHM algorithm markedly improved inter-rater reproducibility of arteriolar morphometric analysis, and SD-OCT may represent a promising noninvasive method for *in vivo* arteriolar morphometry.

## Introduction

Retinal vessels offer a unique opportunity to investigate the relationship between arteriolar characteristics and cardiovascular, and cerebrovascular diseases [1]. Arteriolar narrowing predicts the incidence of hypertension, diabetes, coronary artery disease, and stroke [2–5]. In previous studies, the most common method used for retinal vessel measurement was based on the analysis of images obtained from fundus photography [6]. However, the fundus photography method only yields the retinal vessel caliber diameter, and cannot provide additional morphometric data, such as vessel wall thickness or the wall to lumen ratio (WLR).

Recently, several research groups have utilized spectral-domain optical coherence tomography (SD-OCT) for retinal vessel diameter measurement [7–11]. Commercial SD-OCT can produce a retinal cross-sectional image using A-scans at an axial high-resolution of 7 μm [12]. Retinal vessel structure with vessel wall signal can be observed within the retinal cross section in an optical coherence tomography (OCT) image. Consequently, retinal vessel outer and lumen diameters can be measured for the subsequent calculation of wall thickness [9]. However, most previous studies used manual methods for vessel diameter measurement using software with built-in SD-OCT [8,10,11], or Image J software (National Institute of Health) [7,9]. However, these manual methods are extremely dependent on the subjective visual judgment of the outer and inner vessel wall borders. Our previous study has demonstrated the inter-rater and intra-rater repeatability coefficients of retinal vessel diameter manual measurements in a OCT image were approximately 5 μm [9]. Thus, the manual method may be not accurate enough to detect potential pathophysiological changes in the retinal arteriolar wall, which normally ranges in thickness from 10 to 20 μm [8].

Over the past decade, a microdensitometryapproach has been developed and used in a number of studies for the quantitative assessment of retinal vessel width in a digital fundus image [13–17],where the width of the vessel is estimated at half of the peak height of the intensity profile curve; this approach is also known as width at half maximum (FWHM) [18,19], and it provides a fast and robust estimation of the vessel’s edge, and is less sensitive to noise and adjacent structures than other edge criteria [20]. In this study, to overcome the subjective nature of operator-dependent identification of retinal vessel wall borders, we evaluated the feasibility of the FWHM technique to measure the retinal vessel outer and lumen diameters in an OCT image, and compared the accuracies between manual and FWHM measurements. Lastly, we employ the FWHM approach to evaluate retinal arteriolar morphometric alterations in a population of hypertensive patients.

## Materials and Methods

### Ethics statement

The Ethics Committee of Quzhou People’s Hospital, Zhejiang, China approved the study protocol prospectively. Before study enrollment, all participants gave informed consent to participate in research. Image acquisition, processing, and analysis were performed according to the tenets of the Declaration of Helsinki.

### Study 1

OCT images with a clear retinal arteriolar structure (n = 70), and with a clear retinal venular structure (n = 70) obtained from healthy individuals were randomly selected for analysis in this sub-study. These arterioles and venules were captured from four fundus quadrants. To evaluate the agreement between manual and FHWM methods, rater 1 used the two methods to measure retinal vessel outer and lumen diameters in the same OCT images. The measurements were repeated three times on three different days. To compare the variability of the manual and FWHM methods between different raters, rater 2 performed a fourth measurement using the two methods. Rater 2 was blind to the results from rater 1. Three measurements from rater 1 were averaged for analysis of agreement between FWHM and manual methods. For inter-rater variability, measurement 1 from rater 1 was compared with the results from rater 2. Outcomes were recorded for retinal arteriolar outer diameter (RAOD), retinal arteriolar lumen diameter (RALD), retinal venular outer diameter (RVOD) and retinal venular lumen diameter (RVLD).

### Study 2

We used the FWHM method to explore potential morphometric changes of the retinal arteriole in patients with hypertension. Study patients were randomly recruited from the outpatient clinic of the Quzhou People’s Hospital (Quzhou, Zhejiang Province). Inclusion criteria were an age above 50 years and physician-diagnosed primary hypertension. Exclusion criteria included second hypertension previous or current cardiovascular diseases apart from hypertension history of cerebrovascular disease, myopia more than 6 diopters, significant media opacities, intraocular pressure higher than 21 mmHg, history of glaucoma, uveitis, or retinal disease, or a previous history of laser or intraocular surgery. Age-matched subjects free of hypertension, other cardiovascular or cerebrovascular diseases, and ocular disease were recruited as controls from those who accompanied patients visiting the outpatient clinic. The same operator, who was blind to the clinical characteristics of participants, performed the SD-OCT examination for each participant, and the temporal superior retinal arteriole in the zone B region of the right eye was chosen for scanning. At least ten OCT images of one arteriole were obtained for analysis, and the FWHM measurement of every section was repeated three times. Next, the diameters from the measurements of ten images were averaged for each participant. RAOD and RALD were measured, and morphometric parameters of retinal arteriole were calculated as previously described using the following formulas [21]: wall thickness = (RAOD h RALD)/2, WLR = (RAOD o RALD)/RALD; wall cross-sectional area (WCSA) = t × (RAOD^2^ –RALD^2^)/4.

### Optical coherence tomography imaging

All images were acquired with a Spectralis SD-OCT device (Heidelberg Engineering, Inc., Heidelberg, Germany). Retinal vessel imaging was performed as previously described [9], but with some modification. In brief, retinal arterioles and venules were passed through a region between 0.5 and 1.0 disc distance from the disc margin (zone B) and scanned by an experienced operator. A self-made transparent film was overlaidon the computer monitor to help the operator finding the zone B area in the fundus. Line scan mode was applied, the auto real time was set at 100 frames and the resolution mode was set at high level. The scan line was adjusted manually as perpendicular as possible to the vessel running direction when performing each image capture. The scan was completed before the vessel branching in the zone B region, and only clear images showing the retinal arteriolar or venular wall were considered for further study.

### Image analysis

**Fig 1:**
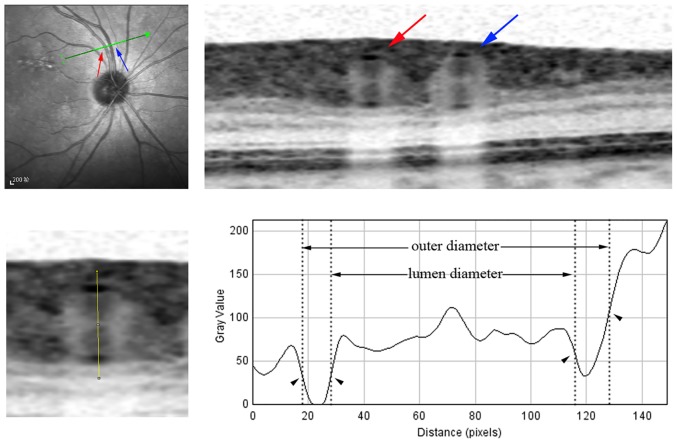
Measurement of the retinal vessel outer and lumen diameters in an optical coherence tomography (OCT) image using the microdensitometry method. (Top left) A line scan was performed across the vessel in zone B, and the line was aligned manually as perpendicular as possible to the running direction of the vessel. (Top right) The cross-sectional structure of the retinal arteriole (red arrow) and venule (blue arrow) could be identified in the OCT image. (Bottom left) The line selection vertically crossed the middle of the upper and lower vessel walls to produce an intensity profile. (Bottom right) The boundary points (arrow heads) were estimated at half maximum intensity for each side of the two parabolas in the profile. The distance between the boundary points was calculated for the vessel outer and lumen diameters.

Before vessel diameter measurement, the vertical-to-horizontal ratio of the OCT image was changed to 1:1 μm, and the image was magnified to the maximum (800%). For the manual method, retinal vessel outer and lumen diameters were manually measured using a caliper tool function of the OCT software (Heidelberg Eye Explorer, version 1.7.1.0). For FWHM-based measurement, magnified OCT images showing the retinal vessel of interest was saved as a BMP file. The saved image was exported to ImageJ (software version 1.47) and the software was used to measure the intensity profile along a line vertically crossing the middle of the vessel (Fig 1). Two parabolas opening upward on the intensity profile represent the upper and lower vessel walls in the OCT image. On the left and right side of each parabola, the maximum and minimum intensity levels were calculated using the average of three consecutive points at the apex and valley. On each side, a fit line was determined by three consecutive points with the largest change of intensity between the first point and third point. Next, the vessel wall boundary point was localized to where the fit line crosses the mid threshold in intensity between the minimum and maximum levels. Lastly, the distances between the boundary points in the two parabolas were calculated for the retinal vessel outer and lumen diameters (Fig 1).

### Statistical analysis

For statistical analysis of the data, we used version 16.0 of SPSS for Windows (SPSS, Chicago, IL, USA). Inter-rater reproducibility was evaluated using an intraclass correlation coefficient (ICC) for each variable measured, coefficient of variation (CV) between raters, and a Bland-Altman plot. The Kolmogorov-Smirnov test was used to analyze all data samples for normality. Comparison of the clinical characteristics between groups was performed using an independent t-test when samples were normally distributed or a Chi-square test when parametric statistics were not possible. Differences in the retinal arteriolar parameters between groups were evaluated using a general linear model. A P*-*value < 0.05 was considered to be statistically significant.

## Results

**Fig 2:**
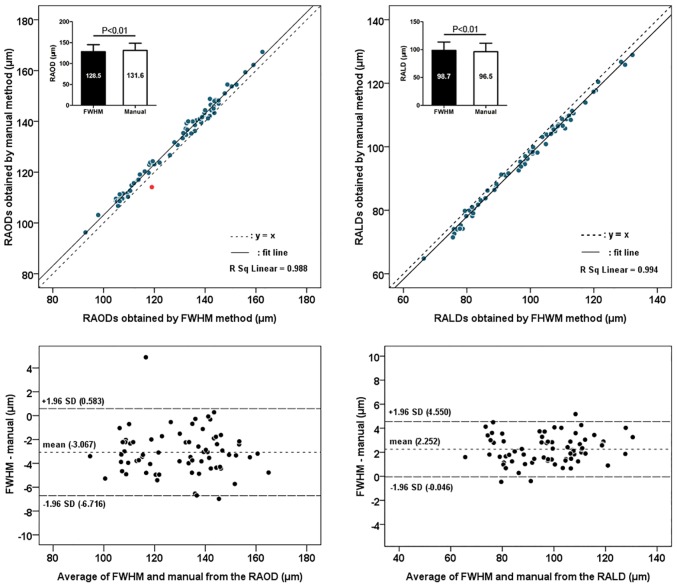
Agreement between manual and full width at half maximum (FHWM) methods for measurement of the retinal arteriolar diameter. RAOD = retinal arteriolar outer diameter; RALD = retinal arteriolar lumen diameter.

**Fig 3:**
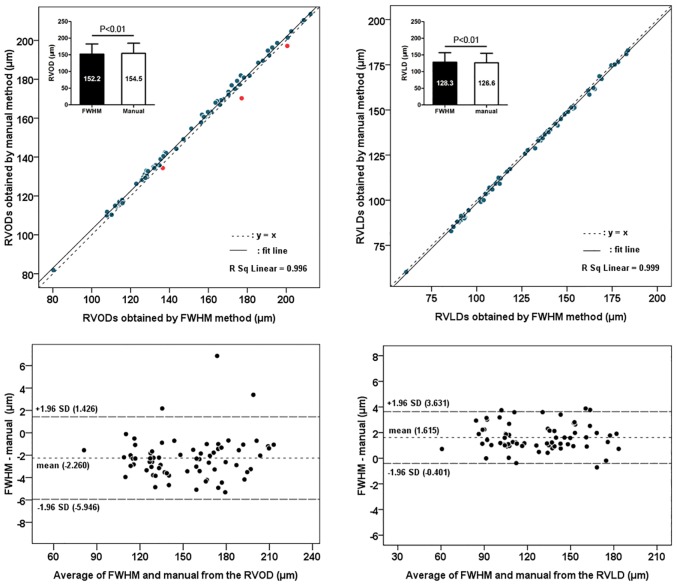
Agreement between manual and full width at half maximum (FHWM) methods for measurement of the retinal venular diameter. RVOD = retinal venular outer diameter; RVLD = retinal venular lumen diameter.

**Fig 4:**
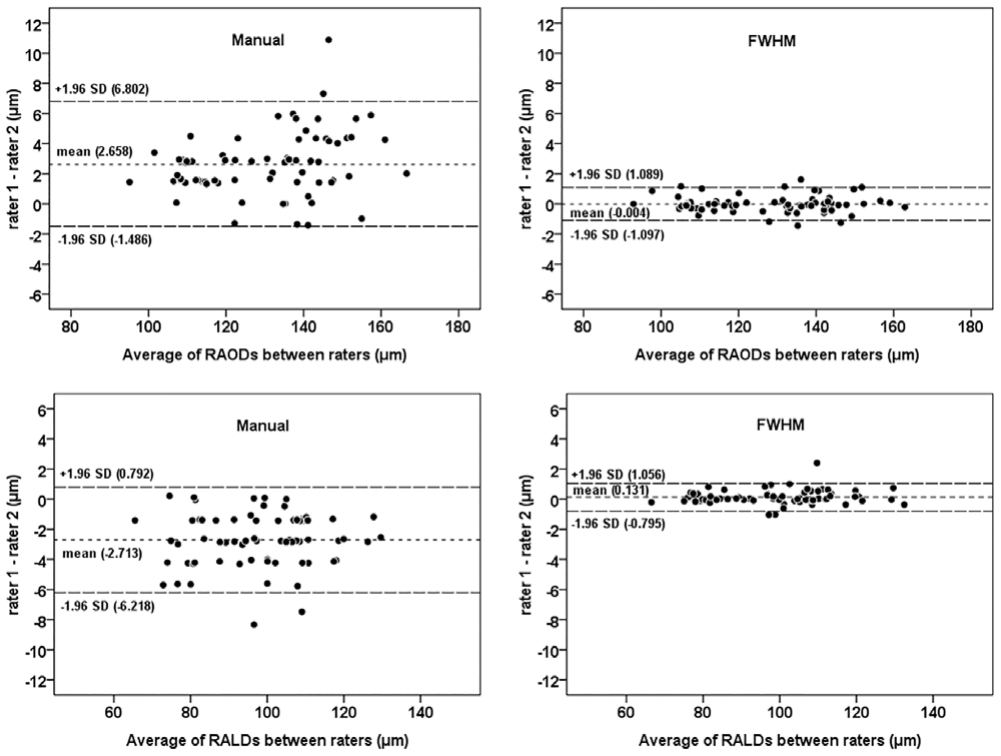
Bland-Altman plots presenting the inter-rater variability of the two methods to measure the retinal arteriolar diameter. Manual (left side); full width at half maximum (FWHM; right side). RAOD = retinal arteriolar outer diameter; RALD = retinal arteriolar lumen diameter.

**Fig 5:**
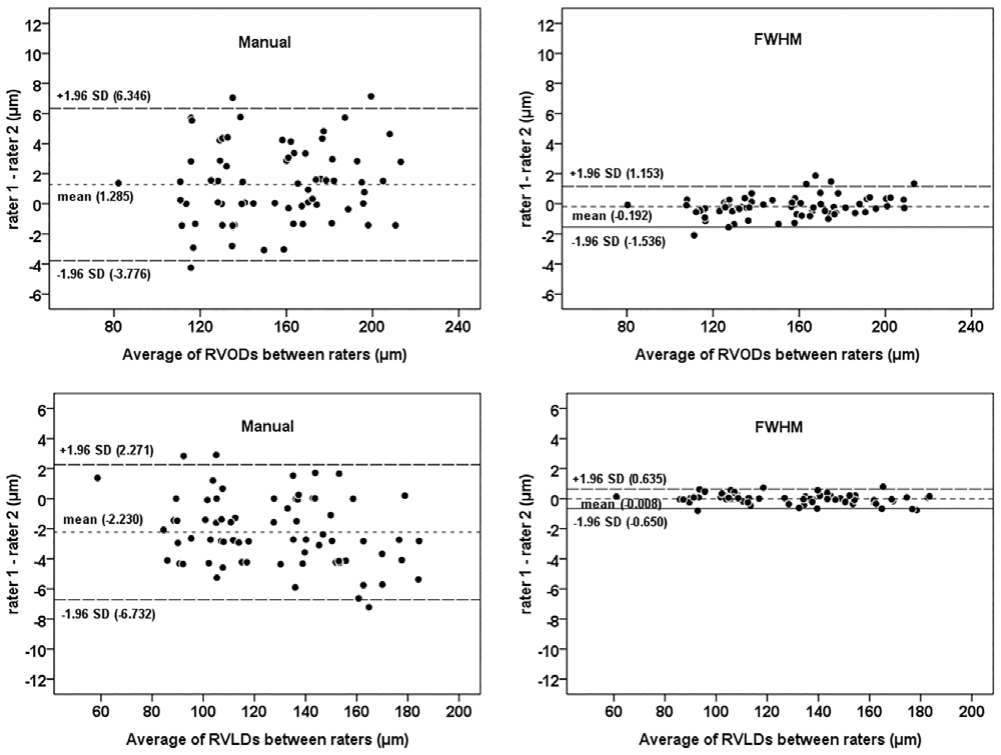
Bland-Altman plots presenting the inter-rater variability of the two methods to measure the retinal venular diameter. Manual (left side); full width at half maximum (FWHM; right side). RVOD = retinal venular outer diameter; RVLD = retinal venular lumen diameter.

In study 1, retinal vessel diameters that were measured using manual and FWHM methods were significantly and highly correlated (all P < 0.001, Pearson correlation) (Figs 2 and 3). Compared with the manual method, the FWHM method identified smaller outer diameters and larger lumen diameters for both retinal arterioles and venules (paired t-test, all P < 0.05). The Bland-Altman plots revealed an agreement between the two measurement methods of the retinal vessel diameters. The 95% limits of agreement for the lumen diameter measurements were narrower than that for the outer diameter measurements (Figs 2 and 3), as the CVs of the differences between two methods were smaller for the lumen diameter measurement (0.521 in RALD and 0.637 in RVLD) compared to those differences for outer diameter measurement (0.607 in RAOD and 0.832 in RVOD). The difference between the two methods for the RAOD measurement was significantly higher than that difference for the RALD measurement (t-test, P < 0.05). Moreover, the difference between two methods for the RVOD measurement was also significantly higher than that difference for the RVLD measurement (t-test, P < 0.05). For the assessment of inter-rater reproducibility, the inter-rater differences, CV values, and the resulting ICCs for each method and the vessel diameters are summarized in Table 1. The inter-rater ICCs were improved when the RAOD, RALD, RVOD, and RVLD were measured using the FWHM approach. The FWHM approach had statistically lower CV measures compared to the manual method for all parameters. The plots in Figs 4 and 5 show the inter-rater agreements for the retinal arteriolar and venular diameter measurements obtained using the manual and FWHM methods. The distances of the 95% limits of agreement for all diameter measurements were much narrower using the FWHM method versus the manual method.

**Table 1:** Inter-rater reproducibility of retinal vessel measurements using manual and FWHM methods.

	Manual			FWHM			
Parameters	Difference (μm, mean ± SD)	CV (%, mean ± SD)	ICC	Difference (μm, mean ± SD)	CV (%, mean ± SD)	ICC	P value*
RAOD	2.66 ± 2.11	1.50 ± 0.94	0.98	-0.01 ± 0.56	0.21 ± 0.22	0.999	< 0.01
RALD	-2.71 ± 1.78	2.04 ± 1.40	0.977	0.13 ± 0.47	0.21 ± 0.26	0.999	< 0.01
RVOD	1.29 ± 2.58	1.05 ± 0.92	0.995	-0.19 ± 0.69	0.25 ± 0.24	1	< 0.01
RVLD	-2.23 ± 2.30	1.49 ± 0.99	0.994	-0.01 ± 0.33	0.13 ± 0.13	1	< 0.01

FWHM = full width at half maximum; CV = coefficient of variation; ICC = intraclass correlation coefficient; SD = standard deviation; RAOD = retinal arteriolar outer diameter; RALD = retinal arteriolar lumen diameter; RVOD = retinal venular outer diameter; RVLD = retinal venular lumen diameter.

*Significance of the comparison of CVs as determined in a Mann-Whitney test.

**Fig 6:**
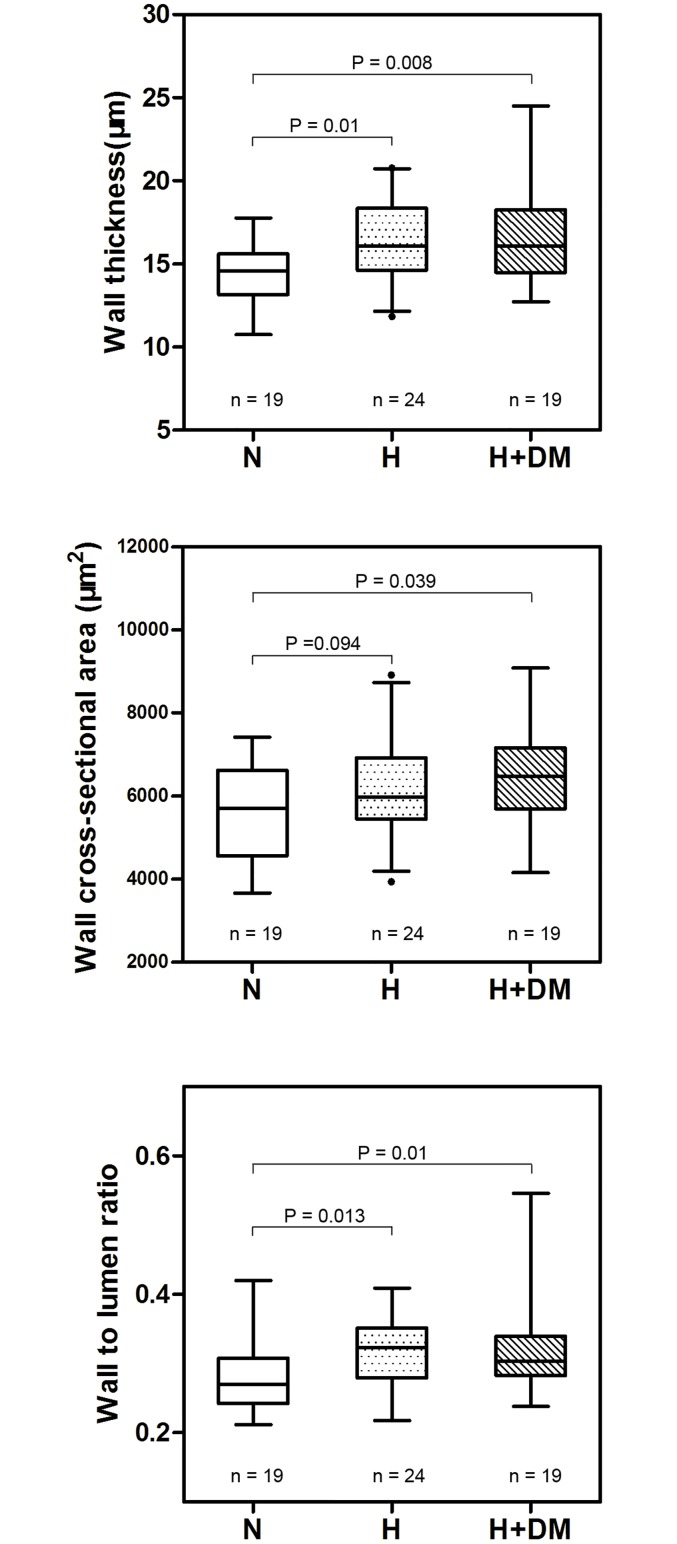
Boxplots showing the distribution of the retinal arteriolar (top) wall thickness, (middle) wall cross-sectional area and (bottom) wall to lumen ratio obtained from normal subjects (N), non-diabetic hypertensive patients (H) and hypertensive patients associated with diabetic mellitus (H+DM). The boxes represent the 25% to 75% interquartile ranges. The horizontal line represents the median values, the bars represent the 5% and 95% confidence intervals, and the dots represent outliers.

**Fig 7:**
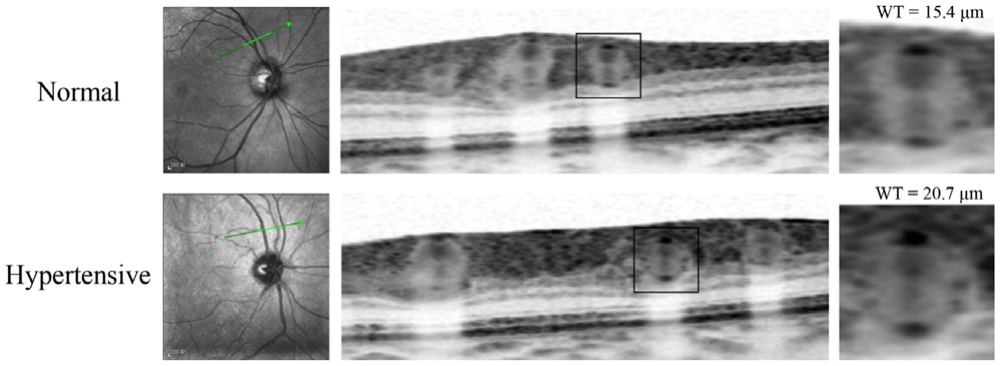
Representative optical coherence tomography images showing the retinal arteriolar structure obtained from (top row) a 63-year-old normotensive female subject and a 66-year-old non-diabetic hypertensive female patient.

Of the 90 eyes that qualified for initial inclusion in this study, five were excluded because of poor image quality, and two patients were unwilling to complete the entire study. Thus the final analysis included the remaining 83 eyes. There were 40 eyes from 40 subjects in the control group and 43 eyes from 43 patients in the hypertensive group. The characteristics of the two groups are presented in Table 2. There were no significant differences noted between the two groups in terms of gender, age, body mass index, smoking history, dyslipidemia and diabetes. Table 3 summarizes the morphometric parameters of the retinal arteriole in the control and hypertension groups. Although RAOD and RALD were not significantly different between the groups, wall thickness, WCSA, and WLR of retinal arteriole were significantly higher in patients with hypertension than in normotensive controls. The statistical results remain unchanged when the characteristics between the two groups were adjusted in a general linear model (Table 3). After considering the differences in vessel branching across individuals, we found that retinal arteriole of 16 eyes (10 control and 6 hypertension) were second branched vessels in zone B. After excluding these 16 cases, 67 eyes remained for further analysis of the first branched arteriole. The results still indicated a thickened arteriolar wall and an increased wall to lumen ratio in hypertension patients (Table 4). We divided the hypertension group into two subgroups, non-diabetic hypertensive and diabetic hypertensive patients. Compared with normal subjects, WT and WLR were significantly increased in both hypertensive patients with and without diabetes. WCSA was again significantly greater in hypertensive patients with diabetes compared with controls; however, no significant difference was found in the WSCA between non-diabetic hypertensive patients and controls (Fig 6). Two example OCT images from normal and hypertensive participants are shown in Fig 7, indicating a arteriolar wall thickening in patients with hypertension.

**Table 2:** Clinical characteristics of the subjects with and without hypertension.

Characteristics	Controls	Hypertension	P value*
Case number	40	43	-
Male sex, n (%)	15 (37.5)	20 (46.5)	0.506
Age (yr), mean ± SD	62.9 ± 5.4	65.1 ± 6.2	0.087
Range	56–82	52–80	-
BMI (kg/m^2^), mean ± SD	23.2 ± 3.1	23.7 ± 2.9	0.422
Current or ex-smoker,n (%)	11 (27.5)	15 (34.9)	0.489
Dyslipidemia, n (%)	12 (30.0)	11 (25.6)	0.807
Diabetes, n (%)	21 (52.5)	19 (44.2)	0.513

BMI = body mass index;

*Age and BMI were analyzed by independent t-test, others were analyzed by Fisher’s exact text.

**Table 3:** Comparison of the morphometric data of the retinal arteriole in the study groups.

Parameters	Controls	Hypertension	*P* value
RAOD (μm)	136.0 ± 2.2	138.4 ± 2.1	0.427
RALD (μm)	106.3 ± 1.9	105.3 ± 1.9	0.685
WT (μm)	14.84 ± 0.36	16.58 ± 0.35	**0.001**
WCSA (μm^2^)	5686 ± 196.5	6375 ± 189.5	**0.014**
WLR	0.283 ± 0.008	0.317 ± 0.008	**0.005**
Adjusted*			
RAOD (μm)	135.9 ± 2.3	138.5 ± 2.3	0.420
RALD (μm)	106.4 ± 2.0	105.2 ± 1.9	0.654
WT (μm)	14.73 ± 0.36	16.68 ± 0.35	**< 0.001**
WCSA (μm^2^)	5647 ± 201.9	6410 ± 194.5	**0.009**
WLR	0.281 ± 0.008	0.319 ± 0.008	**0.002**

RAOD = retinal arteriolar outer diameter; RALD = retinal arteriolar lumen diameter; WT = wall thickness; WCSA = wall cross-sectional area; WLR = wall/lumen ratio.

Data are reported as mean ± standard error. Bold font indicates significant P values.

*Adjusted by gender, age, BMI, smoking history, dyslipidemia, and diabetes with general linear model.

**Table 4:** Comparison of the morphometric data of the first branch retinal arteriole in the study groups.

Parameters	Controls	Hypertension	*P* value
RAOD (μm)	140.0 ± 2.4	139.1 ± 2.2	0.767
RALD (μm)	109.5 ± 2.1	105.7 ± 1.9	0.187
WT (μm)	15.26 ± 0.44	16.67 ± 0.40	**0.019**
WCSA (μm^2^)	6007 ± 228.0	6439 ± 205.3	0.164
WLR	0.283 ± 0.010	0.317 ± 0.009	**0.012**
Adjusted*			
RAOD (μm)	140.3 ± 2.6	138.8 ± 2.3	0.684
RALD (μm)	110.1 ± 2.3	105.2 ± 2.0	0.127
WT (μm)	15.01 ± 0.45	16.80 ± 0.40	**0.009**
WCSA (μm^2^)	5965 ± 241.9	6473 ± 216.4	0.135
WLR	0.278 ± 0.010	0.321 ± 0.009	**0.002**

RAOD = retinal arteriolar outer diameter; RALD = retinal arteriolar lumen diameter; WT = wall thickness; WCSA = wall cross-sectional area; WLR = wall/lumen ratio.

Data are reported as mean ± standard error. Bold font indicates significant P values.

*Adjusted by gender, age, BMI, smoking history, dyslipidemia, and diabetes with general linear model.

## Discussion

A previous study of retinal vessel diameter measurements using fundus photographs, found that the microdensitometry method could underestimate vessel width [13]. Compared with manual method, application of the FWHM method typically resulted in smaller outer diameters and larger lumen diameters. Therefore retinal vessel wall thickness will be smaller using the FWHM method compared to the manual method. Consistent with these observations, we found that the arteriolar wall thickness calculated using the FWHM method was 14.9% smaller than that calculated using the manual method, and 13.9% smaller for the venular wall thickness. In addition, we found that the results were similar when rater 2 performed the intra-rater reliability assessment, showing a smaller outer diameter and a wider lumen diameter using the FWHM method (P <0.05, paired t-test).

Of note, there are still several outliers in the correlation maps (red points in Figs 2 and 3), which indicate that greater retinal outer diameters were obtained using the FWHM method. In the original OCT images of these outliers, we found that the measured retinal vessels were all very close to the retinal surface, the vessel wall and the surrounding tissues (e.g., retinal internal limiting membrane), and thus were difficult to distinguish, and the greater outer diameters took into account the surrounding tissue thickness. Therefore, caution should be used when interpreting these OCT images. However, this issue did not exist in the retinal vessel lumen measurements, because of the good contrast between vessel wall and blood flow.

Our results indicate that the FWHM method significantly improves the precision of measurements for both outer and lumen diameters of retinal arterioles and venules in zone B region. With respect to inter-rater reproducibility, our findings were similar to a report by Flett et al. [22], which showed the FWHM method could provide superior reproducibility. The mean differences in the scatterplots were all nearly zero, showing that there were no systematic discrepancies between the first and second measurements. The limits of agreement in the inter-rater analysis of the FWHM method indicated that differences up to 1.5 μm are possible for larger retinal vessel diameters (Figs 4 and 5). Such differences may be acceptable in clinical trials that detect potential pathophysiological alterations of the retinal vessel diameters.

Patients with higher blood pressure have a narrower retinal arteriolar caliber, and this inverse association between blood pressure and retinal arteriolar caliber has already been demonstrated in different large epidemiological studies using fundus photography analysis, such as the Atherosclerosis Risk In Communities study [2],the Beaver Dam study [23],the Blue Mountains study [24], and Rotterdam study [25]. In present study, we used SD-OCT for retinal vessel diameter measurements, and a narrower retinal vessel lumen diameter was found in hypertensive patients, but this difference did not reach statistical significance. This negative result is probably due to the fact that we evaluated the diameters of only one main retinal arteriole and the results were directly used for comparisons between groups. However, in previous studies using the fundus photography method, summary variables were used for evaluation in hypertension population [2], which were defined as the central retinal arteriolar equivalent (CRAE) and the central retinal venular equivalent (CRVE). The CRAE and CRVE variables were calculated using empirical formulas and respectively represented the size of central retinal artery and vein. Another explanation for the non-significant change in retinal arteriolar lumen diameter can be the small sample size we enrolled in the current study.

In our study, wall thickness, WCSA, and WLR of the retinal arteriole were all significantly higher in patients with hypertension than normotensive controls, even after adjusting for factors such as sex, age, body mass index, smoking, dyslipidemia, and diabetes. The present results are consistent with the common observation that hypertension causes structural changes within the arteriolar wall, often manifesting primarily as a thickened media. To data, few retinal vasculature imaging devices have been used to measure the retinal arteriolar wall. Previous work by Ritt and colleagues using scanning laser Doppler flowmetry reported higher wall thickness and WLR of retinal arterioles in a cohort of never-treated patients with essential hypertension compared to controls, suggesting the important role of blood pressure on retinal arteriolar structure in untreated patients with essential hypertension [21]. Recently, Muraoka et al. [8], using the same SD-OCT as that used in our present study (Spectral HRA + OCT), also demonstrated increased retinal arteriolar wall thickness in a population of hypertensive patients, where their retinal vessel outer and inner diameters were measured manually. Another promising new method, adaptive optics scanning laser ophthalmoscopy, has been used to visualize retinal vascular walls owing to its superior image resolution of the fundus [26]. Koch and associates showed that the WLR of the superotemporal retinal artery was significantly higher in hypertensive patients using adaptive optics imaging and further demonstrate that this condition was positively correlated with mean blood pressure [27]. The outcomes were similar among these studies using different devices, and our results support a previous idea that morphometric parameters of the retinal arteriolar wall can be considered to be more sensitive indicators than retinal arteriolar diameters for retinopathy assessment in hypertension.

Two types of vessel wall remodeling have been described in previous studies, inward eutrophic and inward hypertrophic remodeling, depending on whether the wall cross-sectional area is enlarged [28–31]. Previous studies by Ritt et al. and Koch et al. [21,27], found that the WSCA did not differ between patients with essential hypertension and normotensive patients, suggesting a predominant role of eutrophic remodeling. However in the current study, the mean retinal arteriolar WCSA was significantly greater in the hypertensive group, indicating the predominance of hypertrophic remodeling. Previous *in vitro* studies have shown that eutrophic remodeling is the most common structural change observed in the resistance vessels of individuals with essential (primary) hypertension [28], while hypertrophic remodeling is usually found in hypertension that is associated with diabetes mellitus [32]. Thus, the discrepant result between studies may be due to the difference between patient populations, because nearly half of our hypertensive participants were associated with type 2 diabetes mellitus. When we separately compared non-diabetic hypertensive and diabetic hypertensive patients with controls, the WCSA was unchanged in hypertensive patients without diabetes, but was still significantly increased in hypertensive patients with diabetes. Therefore, our results obtained non-invasively using SD-OCT *in vivo* are in line with previous *in vitro* studies.

Several limitations of this study need to be considered. First, while the FWHM method is less dependent on the operator, there is still a possibility for operator-error because the approach is not fully automated. A second limitation of our study is that currently there is no gold standard method of measurement of retinal vessel structures, and absolute measures of vascular parameters *in vivo* are difficult to obtain. Third, only one main retinal arteriole was chosen for an OCT scan in study 2, and there were no summary variables available such as CRAE or CRVE to estimate the overall retinal vessel wall structure. The temporal superior retinal arteriole seems to be more frequently used for measurement of arteriolar morphology [21,27,33].

## Conclusions

In this study, we introduced and assessed the application of a microdensitometry method using a FWHM algorithm to analyze OCT images. The algorithm shows good agreement with a manual caliper approach but with a significant improvement in the inter-rater reproducibility. Our method has strong potential to be useful for arteriolar morphometric quantification in the clinical setting.

## Supporting Information

S1 File Retinal arteriolar outer and inner diameters data in study 1. (XLS)

S2 File Retinal venular outer and inner diameters data in study 1. (XLS)

S3 File Clinical characteristics and morphometric data of each participant in study 2. (XLSX)
